# Targeting uPARAP with an Antibody–Drug Conjugate Exhibits Efficacy against Mesothelioma and Synergizes with Cisplatin

**DOI:** 10.1158/2767-9764.CRC-25-0381

**Published:** 2026-01-16

**Authors:** Pınar Çakılkaya, Ida Marie Egeland Larsen, Qun Jiang, Kirstine Sandal Nørregaard, Henrik Gårdsvoll, Jingli Zhang, Henrik Jessen Jürgensen, Michaela Hansen Blomquist, Alba Martinez Perlado, Oliver Krigslund, Eric Santoni-Rugiu, Lars Henning Engelholm, Raffit Hassan, Niels Behrendt

**Affiliations:** 1 Finsen Laboratory, Rigshospitalet/Biotech Research & Innovation Centre (BRIC), University of Copenhagen, Copenhagen, Denmark.; 2 Thoracic and GI Malignancies Branch, CCR, NCI, NIH, Bethesda, Maryland.; 3 Department of Pathology, Rigshospitalet, University of Copenhagen, Copenhagen, Denmark.; 4 Department of Clinical Medicine, University of Copenhagen, Copenhagen, Denmark.

## Abstract

Urokinase plasminogen activator receptor–associated protein (uPARAP) is a collagen-internalizing receptor with emerging relevance as a therapeutic target. The involvement of uPARAP in extracellular matrix turnover and stromal remodeling within the tumor microenvironment (TME) makes it an especially promising target in tumors with a high stromal component. Mesothelioma, characterized by its unique complex TME, is a highly refractory cancer type for which no effective targeted therapy exists. uPARAP has been shown to be expressed across all subtypes of mesothelioma, especially in the sarcomatoid and biphasic subtypes, suggesting that a uPARAP-targeted therapy may benefit a specific patient population. In this study, we utilized an antibody–drug conjugate (ADC) comprising an anti-uPARAP mAb conjugated to monomethyl auristatin E via a protease-sensitive linker for preclinical studies on mesothelioma treatment. Using mouse xenograft models with a human mesothelioma cell line or a patient-derived mesothelioma cell isolate, we demonstrated a strong anticancer effect following treatment with the uPARAP-targeting ADC. IHC studies revealed this to be the case, even in tumors exhibiting a heterogeneous expression of uPARAP, suggesting that uPARAP-expressing cancer-associated fibroblasts also play a role in the anticancer effect through a bystander mechanism. Furthermore, we show that combination treatment with cisplatin, commonly used in first-line mesothelioma therapy, leads to a strongly enhanced anticancer effect relative to treatment with either drug alone. Our study demonstrates the potential utility of a uPARAP-targeted ADC as a therapeutic option for mesothelioma, alone or in combination with chemotherapeutics. This perspective is particularly emphasized by a recently initiated clinical trial with a humanized anti-uPARAP ADC for the treatment of other malignancies.

**Significance:**

This study highlights the translational promise of a uPARAP-targeted ADC for the treatment of mesothelioma, a cancer lacking effective therapies. Demonstrating potent preclinical efficacy and synergy with cisplatin, our findings support the clinical advancement of a uPARAP-directed ADC strategy, particularly considering an ongoing clinical trial evaluating this approach in other malignancies.

## Introduction

Mesothelioma is an aggressive and treatment-resistant cancer primarily caused by asbestos exposure. Despite preventive measures, the estimated global burden of mesothelioma in 2022 was approximately 30,000 new cases, with 25,000 deaths (1). The median overall survival (OS) after diagnosis for pleural mesothelioma is only approximately 18 months (all subtypes) in palliative care, with an even lower OS observed in the sarcomatoid subtype (3–6 months; refs. 2–4).

Currently, there is a complete lack of effective treatment options for this disease. Drug resistance poses a very severe obstacle affecting approximately half of the first-line treatments. Currently, this regimen includes cisplatin and pemetrexed (5, 6). Despite recent advancements, including the approval of immune checkpoint inhibitor combinations, which have resulted in improved OS (2), innovative therapeutic strategies are urgently needed.

Antibody–drug conjugates (ADC) are a promising cancer treatment approach that combines a mAb with a cytotoxic payload. ADCs directed against a variety of targets (including HER2, Trop2, tissue factor, folate receptor α, and Nectin-4) have now been approved for the treatment of several malignancies, such as breast, urothelial, gastric, and cervical cancers (7). Yet, no ADCs have been approved for clinical use for the treatment of mesothelioma. Our study addresses this treatment option by evaluating a novel ADC targeting the endocytic receptor urokinase plasminogen activator receptor–associated protein (uPARAP; Endo180, CD280, product of the *MRC2* gene). This receptor is highly expressed in all main subtypes of mesothelioma, especially in sarcomatoid and biphasic mesothelioma (8), and in cancer-associated fibroblasts (CAF; refs. 9–11).

uPARAP is a recycling transmembrane receptor with important roles in the tumor microenvironment (TME). It is involved in extracellular matrix turnover by binding, internalizing, and directing interstitial and basement membrane collagens for intracellular degradation. Through this process, uPARAP contributes to cancer cell invasion and metastasis (9, 12, 13). uPARAP overexpression has also been observed in mesenchymal-like cancer tissues, including glioblastoma multiforme (14), osteosarcoma (15), soft tissue sarcomas (16), as well as in basal-like breast cancer (17). In contrast, in healthy tissues, the expression of uPARAP is low and limited to certain populations of active fibroblasts, subsets of macrophages, chondrocytes, osteoblasts, and osteocytes associated with ossification in developmental age or healing processes (18–20).

In a recent study, we demonstrated a dose-dependent and target-specific elimination of mesothelioma cells with a uPARAP-targeting ADC *in vitro* (8). This ADC was created using the highly potent cytotoxin, PNU159682 (8), which can be used only at low doses *in vivo* (21). In this study, we use a modified anti-uPARAP ADC, based on a clinically validated payload, to study the utility of an ADC-based treatment *in vivo*, using both patient-derived and cell line–based mesothelioma xenograft mouse models with varying uPARAP expression profiles. Furthermore, we examine the combination of the ADC with existing first-line mesothelioma therapy for enhanced therapeutic effect.

## Materials and Methods

### Cell culture

All cells were grown at 37°C with 5% CO_2_, following the culture recommendations from the respective suppliers, as follows: Cells of the biphasic human mesothelioma cell line H‐Meso‐1 (RRID: CVCL_5759; obtained from CLS Cell Lines Service GmbH for *in vitro* work, obtained from the NCI/DCTD Tumor Repository for *in vivo* work) were cultured in RPMI 1640 GlutaMAX medium (Gibco, Life Technologies), supplemented with 1% penicillin–streptomycin (10,000 U/mL, Life Technologies) and 10% FBS (Avantor). The human squamous cell lung carcinoma EBC-1 cell line (RRID: CVCL_2891; JCRB Cell Bank) was cultured in DMEM GlutaMAX medium (Gibco, Life Technologies) supplemented with 1% penicillin–streptomycin and 10% FBS. Epithelioid human mesothelioma ONE58 cells (RRID: CVCL_2671; ECACC) were cultured in RPMI 1640 GlutaMAX medium supplemented with 1% penicillin–streptomycin, 25 mmol/L HEPES buffer (Life Technologies), and 5% FBS. Epithelioid human mesothelioma JL-1 cells (RRID: CVCL_2080; DSMZ) were cultured in DMEM GlutaMAX medium (Gibco, Life Technologies) supplemented with 1% penicillin–streptomycin, 25 mmol/L HEPES (Life Technologies, and 20% FBS. Early-passage mesothelioma cells (epithelial NCI-meso16, NCI-meso21, NCI-meso57, NCI-meso63, NCI-meso77, and biphasic NCI-meso79) were established from ascites or pleural fluid obtained from patients with mesothelioma, treated at the NCI. The methods for establishing primary culture cells have been described previously (22, 23). All primary cells were cultured in RPMI 1640 (Gibco, Life Technologies) supplemented with 2 mmol/L glutamine (Gibco, Life Technologies), 1% penicillin–streptomycin, 1 mmol/L sodium pyruvate (Gibco, Life Technologies), and 20% FBS. To prevent genetic drift, the H-meso-1 cell line that was used for *in vivo* studies was cultured for fewer than six sequential passages after receipt from the above-indicated provider. This cell line and all primary isolates were tested for *Mycoplasma* and other pathogens at the Animal Diagnostic Laboratory for Cancer Research operated by Leidos Biomedical Research, Inc. All other cells were tested for *Mycoplasma* and related pathogens using the IMPACT test (IDEXX BioAnalytics).

### Western blot

Primary cells and H-Meso-1 cells were lysed at 0°C for 20 minutes in NP-40 lysis buffer (Thermo Fisher Scientific) containing cOmplete, Mini, EDTA-free protease inhibitor cocktail (Roche) according to the supplier’s recommendations. Lysates were clarified by centrifuging at 20,000 × *g* for 15 minutes at 4°C; subsequently, protein concentrations were determined using the Pierce BCA Protein Assay Kit (Thermo Fisher Scientific). Protein samples (25 μg) were subjected to nonreducing pretreatment by boiling in NuPAGE LDS sample buffer (Invitrogen) and electrophoresed on Mini-PROTEAN TGX 4% to 15% gradient gels (Bio-Rad Laboratories Inc.). Prestained Plus Protein Ladder (10–250 kDa, Vita Scientific) was used for molecular mass determination. A Mini-PROTEAN Tetra Cell (Bio-Rad Laboratories) was used for SDS-PAGE and wet electroblotting onto polyvinylidene difluoride membranes (2 hours, 100 V). Then, membranes were blocked with 5% nonfat milk powder (RPI, Research Products International) in TBS with 1% Tween 20 detergent (TBS-T) and incubated overnight at 4°C either with 0.5 μg/mL anti‐uPARAP mAb clone 2.h.9:F12, (Millipore, RRID: AB_11213255) or 0.5 μg/mL β-actin mAb (antibody against house-keeping gene product for normalization; Thermo Fisher Scientific, RRID: AB_2536844) in 1% TBS-T with 2% BSA (Sigma-Aldrich). Incubation was done on a shaking platform after cutting the blots at the designated size level to be incubated with antibodies separately. After washing, probing was done by incubation with 4 μg/mL goat anti‐mouse Alexa Fluor Plus 800–coupled antibody (Thermo Fisher Scientific, RRID: AB_2633279) for 30 minutes at room temperature. Blots were imaged with the Odyssey CLx fluorescence imaging system (LI‐COR Biosciences) using Image Studio Software (version 5.2). Densitometry of all blots was performed using Image Studio Lite (version 5.2.5) software. The assay was repeated in three independently generated runs with two separate biological replicates.

### Antibody production

Anti-uPARAP mAb 9b7 (subtype IgG1 Kappa; ref. 8) for fluorescence labeling and ADC construction was produced recombinantly using the ExpiCHO-S Expression System Kit (A29133, Thermo Fisher Scientific) following the manufacturer’s recommendations and the procedure described previously (24). In brief, vectors for transfection (produced by VectorBuilder GmbH) were constructed by cloning DNA sequences encoding the variable IgG regions of the mAb 9b7 heavy and light chains into expression vectors encoding the constant regions of murine IgG heavy and light chain sequences, respectively. The resulting IgG1 antibody was purified on HiTrap protein G HP columns (Merck) using Äktapurifier (GE Healthcare). Protein homogeneity was confirmed through size-exclusion chromatography on a Superdex 200 Increase column 10/300 GL (GE Healthcare). The IgG concentration was determined by absorption spectroscopy, utilizing A_280_ = 1.4 for an IgG concentration of 1 mg/mL. The nontargeting, isotype-matched control mAb directed against trinitrophenol, aTNP, and anti-uPARAP mAb 9b7 (8) for initial ELISA studies were produced using hybridoma cell culture protocols described in (10, 25) and purified in the same manner.

### Cellular uptake studies: confocal microscopy and flow cytometry

For confocal microscopy, cells were cultured in 35-mm glass‐bottom culture dishes (P35G‐0‐14‐C, MatTek *In Vitro* Life Science Laboratories), pretreated with type-1 collagen (0.3 mg/mL; 354236, Corning). A total of 10,000 NCI-Meso79 or H-Meso-1 cells were seeded onto the cover glass of the culture dishes in their respective complete culture media at 37°C with 5% CO_2_. After cell attachment, the media were exchanged with media supplemented with 2 μg/mL AF647-labeled 9b7 antibody, AF647‐labeled aTNP, or with no antibody. The cells were then cultured under the same conditions overnight to allow antibody endocytosis. Next, cell surface staining was performed using a 1:500 dilution of wheat germ agglutinin Alexa Fluor 488 conjugate (Thermo Fisher Scientific). Cell nuclei were counterstained with Hoechst stain 33342 (H3570, Thermo Fisher Scientific), also used at a 1:500 dilution. Cell examination and image acquisition were conducted using a Zeiss LSM 800 confocal microscope, followed by image processing with the Zen system (Zeiss, 3.2).

For flow cytometry analysis, cells were seeded in 24-well plates (10^5^/well) in duplicates in their respective media and cultured overnight. Fluorescence-labeled mAbs (final concentration 2 μg/mL) were then added to the cells. In a parallel set of wells, cells underwent uPARAP depletion using the anti-uPARAP mAb 5f4 (26). For these wells, cells were preincubated with 20 μg/mL mAb 5f4 for 1 hour, and the same concentration of mAb 5f4 was included in the assay medium during incubation with the fluorescence-labeled mAbs. After overnight incubation at 37°C, cells were detached using 0.25% trypsin-EDTA with proteinase K (50 μg/mL). The cells were then centrifuged at 500 × *g* for 2 minutes at 4°C and resuspended in 2% FBS in PBS. Finally, 10,000 cells were analyzed on a Fortessa flow cytometer (BD Biosciences). For each cell line, intact single cells were identified using forward and side scatter parameters, effectively eliminating cell debris and cellular aggregates before assessing internalized fluorescence. Internalized mean fluorescence intensity (MFI) was calculated by subtracting control sample values (cells without antibody addition) from the test sample MFI value. Data were analyzed using FlowJo software (RRID: SCR_008520).

### ADC preparation

For the preparation of ADCs, the 9b7 and aTNP mAbs were conjugated to monomethyl auristatin E (MMAE) through cysteine-directed coupling, using a commercial maleimide valine–citrulline (vc)-MMAE linker toxin derivative (mc-vc-PAB-MMAE, MedChemExpress). The conjugation followed the previously described methods (24). In brief, antibodies at a concentration of 3 mg/mL underwent partial reduction of disulfide bonds by incubation with 2.5 mmol/L dithiothreitol (DTT) in 50 mmol/L sodium borate and 50 mmol/L NaCl buffer (pH 8.0) for 30 minutes at 25°C on an Infors HT Unitron incubator shaker at 150 rpm. This was followed by buffer exchange to PBS pH 7.4 with 1 mmol/L EDTA, using two Zeba Spin Desalting Columns (Thermo Fisher Scientific) to ensure the complete removal of DTT. The linker toxin reagent was dissolved in an 80/20 (v/v) propylene glycol/*N,N*-dimethylformamide (PG/DMF) solution and added to the partially reduced antibodies at a fivefold molar excess. During conjugation, the final concentrations of PG and DMF were adjusted to 20% and 5%, respectively. The reaction mixture was incubated for 2 hours at 25°C on the incubator shaker as described above. ADCs were subsequently purified by running the sample over two Zeba Spin Desalting Columns to eliminate any uncoupled linker toxin.

### ADC characterization

For qualitative assessment of the conjugation, the modified antibodies were visualized by SDS-PAGE under reducing conditions to reveal a shift of the electrophoretic mobility of the IgG heavy chain, as also shown previously (24). To do that, the ADCs were treated with 40 mmol/L DTT in 1X NuPAGE LDS sample buffer (Invitrogen) and incubated at 100°C for 5 minutes to reduce disulfide bonds. Then, 1 μg samples (9b7 and aTNP-based ADCs) were loaded onto NuPAGE BOLT 4% to 12% Bis-Tris Plus gels (Thermo Fisher Scientific) and separated by SDS-PAGE, followed by staining with Coomassie Brilliant Blue. The protein concentration and the molar drug-to-antibody ratio (DAR) of each ADC were determined by UV/Vis absorption spectroscopy (QIAxpert, QIAGEN). For DAR determination, absorbance was measured at 248 and 280 nm, after which the extinction coefficients for MMAE and IgG at both wavelengths (27) were used for calculation according to the average DAR calculation method (28). Average DARs of 4.1 and 4.3 were achieved for 9b7-MMAE and aTNP-MMAE, respectively.

Studies on the binding affinity of uncoupled mAb 9b7 and 9b7-MMAE toward uPARAP were done in an ELISA system, performed as described in (29) with minor modifications. The immobilized antigen was a recombinant uPARAP fusion protein comprising the 10 extracellular domains (10). Briefly, the wells of microtiter plates (Nunc-Immuno Maxisorb F96, Thermo Fisher Scientific) were coated with 50 ng of recombinant uPARAP. After blocking and wash, incubation was performed with a dilution series of test antibodies (uncoupled mAb 9b7 or 9b7-MMAE) overnight at 4°C. For quantification of bound antibody, after renewed washing, wells were incubated with polyclonal rabbit anti-mouse immunoglobulin/horseradish peroxidase (HRP; Dako; 1.3 g/L; RRID: AB_2636929), used at 1:2000 dilution. This was followed by wash and detection of HRP activity using 3, 3′, 5, 5′-tetramethylbenzidine chromogenic substrate (Kementec) and recording of absorbance at 450 and 540 nm, using the latter wavelength for background subtraction.

### Assay for cellular sensitivity to ADCs and synergistic score

H-Meso-1 cells or mesothelioma cells from the primary isolates were seeded in 100 μL of their respective complete growth media in tissue culture–treated 96-well plates at 3,000 cells/well in triplicate. After overnight incubation at 37°C with 5% CO_2_, culture media were replaced with 100 μL of respective fresh medium containing 9b7-MMAE or aTNP-MMAE ADCs, added as a 1:4 dilution series. After 6 days of incubation, the plates were analyzed using 3-(4,5-dimethylthiazol-2-yl)-5-(3-carboxymethoxyphenyl)-2-(4-sulfophenyl)-2H-tetrazolium (MTS) cell viability assay. This was performed by adding 15 μL of CellTiter 96 AQueous One solution reagent (Promega) to each well, followed by incubation for 1 hour. Subsequently, the plates were read in a plate reader at 490 and 630 nm, using absorption at the latter wavelength for background subtraction. Relative cell viability was calculated by setting the non–ADC-treated control wells at 100% viability. The effect of free MMAE, used in concentration series, was tested in the same manner, including the additional mesothelioma cell lines ONE58 and JL-1 and the squamous cell lung carcinoma cell line EBC-1. In this case, relative cell viability was determined by comparison with DMSO-treated control wells (used at the same final concentration as that in the MMAE-treated samples), set to 100% viability.

For studies on combination treatment *in vitro*, experiments with NCI-Meso79, H-Meso-1, ONE58, and JL-1 cells were conducted in the same manner but by incubating cells with varying concentrations of 9b7-MMAE and cisplatin in combination. The analyzed combined concentrations covered intervals from 0.002 to 40 μg/mL for 9b7-MMAE and from 0.002 to 10 μg/mL for cisplatin, respectively. A total of 49 combinations and mono treatment series (seven concentrations) were analyzed in triplicate for all cell isolates/cell lines studied. Cell survival was recorded for all samples as above. Synergy is defined as the observed effect of combination therapy exceeding the total effect of the monotherapies. The data obtained were then analyzed for calculation of the synergy score using the ZIP scoring approach (30) with the SynergyFinder Plus web application for interactive analysis of multidrug combination data (https://synergyfinder.org/#!/).

### *In vivo* tumor models

For the H-Meso-1 model, female NOD.Cg-*Prkdc*^scid^ Il2rg ^tmlWjl^/SzJ; RRID: IMSR_JAX:005557 (NSG) mice were acquired from The Jackson Laboratory (The JAX Cancer Center, Frederick National Laboratory for Cancer Research). Five-week-old mice (weighing 18–25 g) were implanted with 5 × 10^6^ H-Meso-1 cells in saline in the flank under isoflurane anesthesia. When tumors had developed, the mice were treated intravenously via tail vein injection on days 0, 4, and 7 with 9b7-MMAE, nontargeted aTNP-MMAE, unconjugated anti-uPARAP 9b7 mAb, or saline (0.9% sodium chloride). ADCs and the unconjugated mAb were administered at 6 mg per kg of mouse weight for each injection. The saline group received the equivalent volume as the other groups based on their body weight. The mice were euthanized with CO_2_ inhalation based on endpoints: tumor volume reaching 1,000 mm^3^ or necroptotic regions visually accounting for more than 40% of the tumor area.

For the patient-derived xenograft model, female NSG mice were obtained from Charles River Laboratories. The model was established by injecting 8 × 10^6^ NCI-Meso79 cells resuspended in PBS mixed with 50% Matrigel (Discovery Labware) into the flank. Cell injections were conducted under Zoletil anesthesia with Carprofen. After the tumor had been established, mice were treated on days 0, 4, and 7 with 9b7-MMAE, nontargeted aTNP-MMAE, PBS, cisplatin (cis-diaminodichloroplatinum; HY-17394, MedChemExpress), or combination treatment with 9b7-MMAE and cisplatin. Injections were administered via the tail vein, except for cisplatin, which was given through the intraperitoneal route. ADCs and cisplatin were administered at doses of 6 and 1 mg/kg, respectively, for each injection. Humane endpoints were predetermined as tumor length reaching 12 mm or a maximum weight loss of 15%.

Tumor length and width were measured using an electronic caliper, and tumor volume was calculated using the following formula: V = (L × W^2^) × 0.4. All mice were terminated because of the tumor length and size endpoints. Following termination, intracardiac perfusion with 10 to 20 mL PBS was performed, and the tumors were harvested, divided into two groups for middle tumor-sectioning, and prepared for histologic analysis.

### Histologic analyses

The tumors (minimum of three from each group) were fixed in 10% PBS for 2 days at 4°C, processed (ASP300S, Leica), paraffin-embedded (Tissue-Tek TEC 5 Tissue Embedding Console System), and sectioned on a water bath microtome for 3.5 μm midtumor cross-sections. Hematoxylin and eosin (H&E) staining was then performed on preheated sections (30 minutes at 60°C) using HE Gemini (Axlab) with H&E (Histolab Products AB). The uPARAP and species-specific murine lamin B1 stainings were performed using Bond RX (Leica) on preheated parallel sections using the Bond Polymer Refine Detection Kit (Leica) without the post-primary reagent. In brief, the slides were deparaffinized for an additional 30 minutes at 60°C, incubated with dewax solution (Leica) for 30 seconds prior to a 5-minute incubation with peroxide block, and underwent antigen retrieval with Bond epitope retrieval solution (ER2, Leica) for 10 minutes at 100°C. The slides were incubated with 150 μL of 0.1 μg/mL anti-uPARAP antibody (Rec. rabbit 2H9F12, Finsen Laboratory; ref. 24) for 15 minutes, followed by the Bond anti-rabbit HRP polymer–conjugated secondary antibody (RRID: AB_3659616, R&D Systems) for 8 minutes. Afterward, the slides were incubated with DAB Refine for 10 minutes, followed by a 5-minute incubation with hematoxylin for nuclear counterstain. The same protocol was followed for the murine lamin B1 stain using 50 μg/mL rabbit anti-mouse lamin B1 antibody (HistoSure, Synaptic Systems, RRID: AB_3677699), except additional washing steps were added after the primary antibody incubation for all sections, and sections from H-Meso-1 tumors underwent 30 minutes of antigen retrieval. All slides were dehydrated on HE Gemini, air-dried for 20 minutes, and mounted with Tissue Mount (Sakura Finetek). All slides were scanned (Nanozoomer, Hamamatsu) and analyzed by three independent observers (ESR, PC, and IMEL) in a blinded manner, reaching common consensus in cases of discrepant initial findings.

### Statistical analyses

The statistical analyses were performed using GraphPad Prism version 10 (RRID: SCR_002798). The Mantel–Cox log-rank test was used to compare Kaplan–Meier survival curves.

### Ethics

Patient-derived cell lines were established from ascites or pleural fluid obtained from patients with mesothelioma treated at the NCI on an Institutional Review Board–approved Mesothelioma Natural History protocol (ClinicalTrials.gov NCT 01950572). The study was conducted in accordance with the principles of the International Conference on Harmonization–Good Clinical Practice guidelines. Animal experiments performed at the NIH were conducted under NIH guidelines and approved by the NCI Animal Care and Use Committee under protocol number LMB-053. The other part of the animal experiments was performed with permission from the Danish Veterinary and Food Administration, under permit number 2020-15-0201-00533 (L.H. Engelholm; studies conducted at the Finsen Laboratory/BRIC), in accordance with the guidelines of the Declaration of Helsinki.

## Results

### Initial treatment studies using H-Meso-1, a cell line–derived mesothelioma model

**Figure 1.**
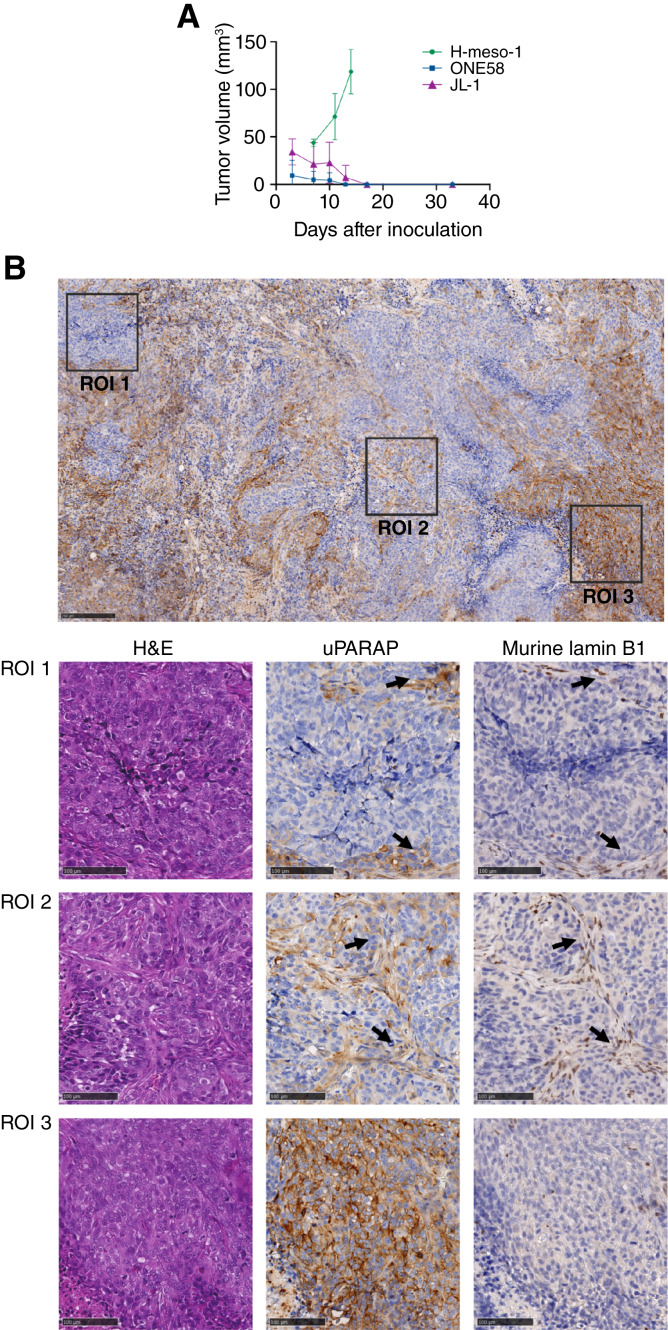
Characteristics of the H-Meso-1 tumor model. **A,** Growth curves for H-Meso-1, ONE58, and JL-1 cells. Cells were inoculated subcutaneously into the flanks of NSG mice at a density of 5 million cells in 100 μL respective media for all three cell lines, after which tumor growth was followed by palpation. ONE58 cells shrank and became undetectable in 7–13 days, whereas JL-1 cells disappeared 13–17 days after inoculation (no growth observed until day 35). H-Meso-1 tumors remained viable and showed consistent growth, providing treatable models within 15 days. **B,** Histologic analysis of H-Meso-1 tumors after growth in mice. Top, IHC staining for uPARAP in a large tumor area (example from a tumor cross-section from a tumor-bearing mouse after saline-treatment; see Fig. 2. Scale bar: 250 μm). The field includes multiple areas with varying stromal infiltration. The staining shows heterogeneous uPARAP expression throughout the tumor. Bottom, enlarged views of selected smaller tumor regions from the top (scale bars, 100 μm). Left, H&E staining; middle, uPARAP staining; right, mouse-specific lamin B1 staining. Region of interest (ROI) 1: uPARAP-negative cancer cells, surrounded by uPARAP-positive fibroblast cells. ROI 2: uPARAP-positive fibroblasts present within a uPARAP-negative cancer region (arrows show infiltrating fibroblasts). ROI 3: uPARAP-positive cancer area devoid of fibroblast infiltration.

As we have previously demonstrated the expression of uPARAP in three human mesothelioma cell lines *in vitro* (8), we first examined their capability to form solid subcutaneous tumors in NSG mice, enabling ADC treatment studies *in vivo*. It turned out that one cell line, H-Meso-1, could indeed form growing tumors, reaching a tumor size of 100 mm^3^ within 2 weeks. The other two cell lines, ONE58 and JL-1, failed to establish growing tumors in mice (Fig. 1A).

**Figure 2.**
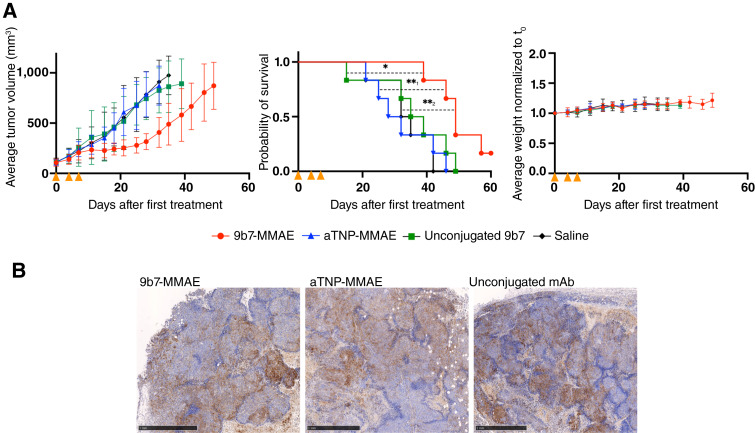
Treatment of H-Meso-1 tumors *in vivo*. **A,** H-Meso-1 cells were injected into the flank of NSG mice (*n* = 6 in each treatment group). Tumors were allowed to grow until a volume of 80–150 mm^3^. 9b7-MMAE ADC (red), non-targeted aTNP-MMAE ADC (blue), or unconjugated mAb 9b7 (green) were administered at 6 mg/kg in intravenous injections on days 0, 4, and 7 (orange arrowheads). A group of saline-injected mice (black) was used as a vehicle control. Left, average tumor volume in treatment groups (curves are shown using the last observation carried forward until 50% of the mice had reached the humane endpoint); middle, Kaplan–Meier curves showing survival for each treatment group with log-rank test (all survival curves being limited by the experiment’s predefined humane endpoints); and right, average mouse weight in groups. Grouped data are shown as the mean ± SD. *, *P* = 0.03; **_1_, *P* = 0.006; **_2_, *P* = 0.004. **B,** Representative histologic comparison of uPARAP staining in H-Meso-1 tumors treated with indicated drugs (for comparison with saline, see Fig. 1B). Scale bars, 1 mm. Similar expression levels with heterogeneous profiles and comparable fibroblast infiltration were observed in all H-Meso-1 tumors.

Therefore, a mouse xenograft model using the H-Meso-1 cell line was set up for initial treatment studies with an anti-uPARAP ADC (Figs. 1 and 2). In these experiments, mice were randomized into treatment groups once the tumor volume had reached 80 to 150 mm^3^ and treated by intravenous injections with ADCs or control reagents. The anti-uPARAP ADC used in these studies has been characterized previously (24) and is based on the mAb 9b7 (8), conjugated with the clinically validated payload MMAE (31) through a cleavable vc linker. mAb 9b7 is a murine IgG reactive with both human and murine uPARAP (24). This ADC, 9b7-mc-vc-PAB-MMAE (in the following designated 9b7-MMAE), binds to uPARAP with an affinity equal to that of the uncoupled antibody (Supplementary Fig. S1A). 9b7-MMAE was used along with a negative control ADC comprising an irrelevant mAb (anti-trinitrophenol, aTNP) and the same cytotoxin; see (24) and Supplementary Fig. S1B for electrophoretic characteristics of both ADCs.

A histologic examination of excised H-Meso-1 tumors, taken from mice not subjected to ADC treatment, revealed mesothelioma cells arranged in characteristic confluent islands. These islands displayed great variability in uPARAP expression across different tumor regions [Fig. 1B (top)]. A high degree of mouse stromal cell infiltration was observed, as identified by utilizing an antibody specific for murine cells [anti-mouse nuclear lamina protein, lamin B1; Fig. 1B (bottom)]. These murine stromal cells were strongly positive for uPARAP with consistent staining throughout the tumor (Fig. 1B, ROI 2), whereas the tumor cell islands comprised both uPARAP-expressing and uPARAP-negative tumor cells (ROI 1 and ROI 3).

The treatment of the tumor-bearing mice included three doses (d0, d4, and d7) of 6 mg/kg uPARAP-targeting ADC, the control ADC, or unconjugated mAb 9b7 (Fig. 2A). A group of saline-injected mice served as vehicle control. Measurement of tumor growth in the treatment groups revealed an evident therapeutic effect of the uPARAP-targeted ADC [Fig. 2A (left); for individual mice, see Supplementary Fig. S2]. Treatment with this ADC led to growth arrest, although tumor growth resumed at variable rates after day 21. This delay in tumor growth resulted in a reduced tumor volume compared with all control groups, with the average volume being approximately 40% of that in the saline group at day 32. Neither unconjugated mAb 9b7 nor the nontargeted ADC notably affected tumor growth. The uPARAP-targeted ADC also significantly prolonged mouse survival, as noted in Kaplan–Meier plots compared with all controls [Fig. 2A (central)]. Importantly, no difference in the weight of the mice between the groups was observed throughout the experiment, indicating that the ADC treatment was well tolerated [Fig. 2A (right)].

To investigate any potential downregulation of uPARAP after ADC treatment, we also performed IHC analyses on H-Meso-1 tumor sections after treatment. Interestingly, no treatment-induced expression differences were observed, as both the uPARAP expression levels and the aforementioned heterogeneity were indistinguishable in tumors following treatment with either 9b7-MMAE, unconjugated mAb 9b7, or aTNP-MMAE (Fig. 2B).

### Treatment of tumors established from a patient-derived mesothelioma cell isolate

**Figure 3.**
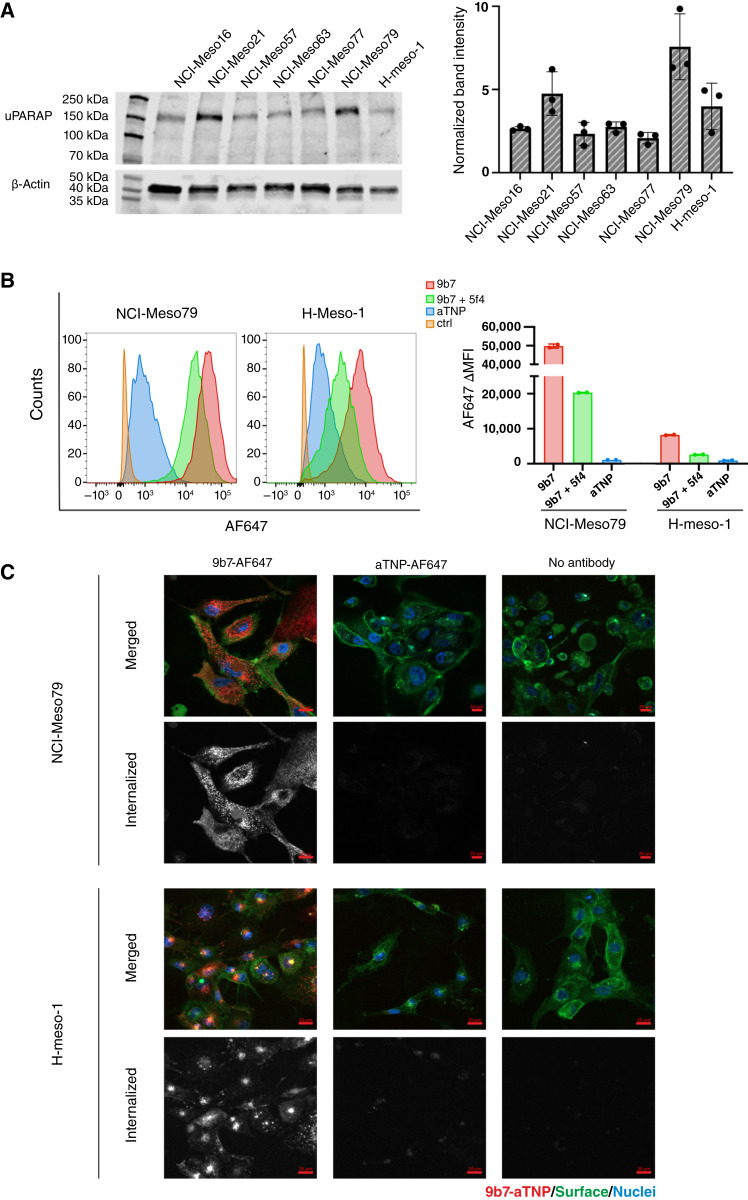
uPARAP expression in primary cell isolates from patients with mesothelioma. **A,** uPARAP expression in primary cell isolates and H-Meso-1 cells analyzed by WB. Left, representative image of uPARAP band detection. Membranes were probed with anti-uPARAP mAb 2h9 (at 180 kDa, top) and housekeeping gene product anti–β-actin antibody (at 42 kDa, bottom) in the same gel. Right, relative uPARAP expression levels, based on the band intensities of the 180 kDa bands after normalization to those of the housekeeping protein in three independent experiments (mean ± SD). **B,** Anti-uPARAP mAb internalization by NCI-Meso79 cells. The uptake of AF647-conjugated anti-uPARAP mAb 9b7 by NCI-Meso79 cells was compared with that obtained in the H-Meso-1 cell line, analyzed by flow cytometry. Left, histograms show the differential uptake of AF647-9b7 (red), AF647-aTNP (blue), and AF647-9b7 after preincubation with anti-uPARAP mAb 5f4 (green). As background controls, samples without antibody addition (ctrl) are shown in orange. Right, bar chart displaying the MFI ± SD of histograms as shown to the left; *n* = 2. **C,** Confocal microscopy imaging of cells after antibody endocytosis. NCI-Meso79 and H-Meso-1 cells were examined after the uptake of mAbs against uPARAP and aTNP (both conjugated to AF647; red). A sample without antibody (no mAb) is shown as an autofluorescence control. All samples were stained with wheat germ agglutinin Alexa Fluor 488 conjugate to image the cell surface (green) and Hoechst stain 33342 for the cell nuclei (blue). For each cell type, the bottom panel displays the internalized fluorescence signal (AF647) in a greyscale range indicator representation. Scale bar, 20 μm.

The antitumor effect observed in the initial study with the H-Meso-1 cell line prompted us to extend the studies to models based on primary tumor cell isolates from patients with mesothelioma. First, we used Western blotting (WB) to explore uPARAP expression in six early-passage mesothelioma cell isolates [Fig. 3A (left)]. An approximate ranking of protein expression levels, based on WB band intensities, pointed to the biphasic mesothelioma subtype–derived NCI-meso79 cells as the isolate having the highest expression of uPARAP. Epithelioid-type mesothelioma cells (NCI-Meso16, NCI-Meso21, NCI-Meso57, NCI-Meso63, and NCI-Meso77) displayed varying uPARAP levels, mostly close to or slightly higher than H-Meso-1 cells, used for comparison [Fig. 3A (right)].

Subsequently, we examined the internalization of fluorescence-labeled anti-uPARAP antibody by NCI-Meso79 cells using flow cytometry, comparing these cells with H-Meso-1 cells, which have been characterized in this respect previously (Fig. 3B; ref. 8). The NCI-Meso79 cells had higher levels of endocytosis than the H-Meso-1 cells and displayed a narrow peak in the histogram, indicating a rather uniform uPARAP expression.

An experiment with live imaging of NCI-Meso79 cells, as well as H-Meso-1 cells studied for comparison, was then performed using confocal microscopy. Imaging cells after exposure to fluorescence-labeled anti-uPARAP mAb confirmed the cellular uptake of the labeled antibody and demonstrated its accumulation in intracellular vesicles (Fig. 3C), consistent with localization in endosomes and lysosomes (32). No cellular uptake was noted with a fluorescence-labeled nontargeted mAb (aTNP) under identical imaging conditions for NCI-Meso79 cells, nor for the comparator H-Meso-1 cells (Fig. 3C).

**Figure 4.**
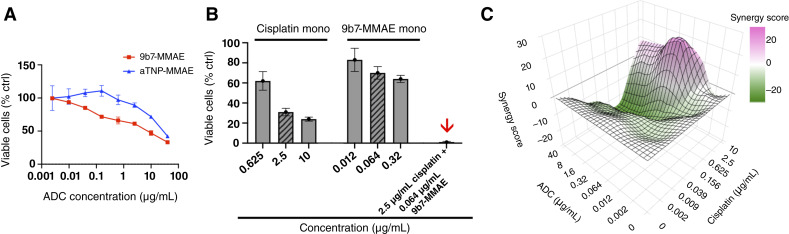
Treatment of NCI-Meso79 cells *in vitro*. **A,** Sensitivity of NCI-Meso79 cells to 9b7-MMAE ADC. The viability of cells is shown after 6 days of cultivation in the presence of varying concentrations of 9b7-MMAE (red) or aTNP-MMAE (blue). Percentages are presented relative to untreated control cell populations as the mean ± SD (*n* = 3). **B** and **C,** Combination treatment of NCI-Meso79 cells with 9b7-MMAE and cisplatin *in vitro*. Cells were cultured for 6 days in the presence of varying concentrations of either reagent alone or in combination as indicated, comprising a total of 49 conditions, followed by measurement of cell viability by MTS assay. **B,** Selected combinations of reagent concentrations show the effect on cell viability of either reagent alone in series or in combination. The combination of 2.5 μg/mL cisplatin and 0.064 μg/mL 9b7-MMAE (arrow) resulted in 0.9% viability and gave a ZIP synergy score of 21, suggesting a response 21% higher than the model-based prediction in the absence of synergy. **C,** Synergy scores for 9b7-MMAE and cisplatin combinations shown in B for the effect on NCI-Meso79 cells, according to the ZIP model. The 3D graph represents all combinations of reagent concentrations, in which a combination is considered synergistic if the score is above 10 and additive between 10 and −10. Scores above 10 were found in the ADC interval from 0.06 to 40 μg/mL when combined with cisplatin concentrations above 2.5 μg/mL (*n* = 3). All dose–response data, along with calculated synergy scores, are presented in Supplementary Fig. S6.

We then examined the sensitivity of all mesothelioma cell isolates to the uPARAP-targeted ADC *in vitro*. A concentration-dependent cell eradication was observed with all isolates (Fig. 4A for NCI-Meso79 and Supplementary Fig. S3 for other isolates), although with notable differences in the concentration dependence and specificity window relative to the negative control ADC. NCI-Meso77 and NCI-Meso79 cells exhibited the highest sensitivity to the uPARAP-targeted ADC while being largely insensitive to the control ADC at concentrations below 1 μg/mL. The sensitivity of these cells was also markedly higher than that of H-Meso-1 cells, included for comparison (Supplementary Fig. S3).

Although the modest sensitivity observed in some cell lines was probably due to low uPARAP expression levels, another contributing factor could be the cellular sensitivity to the free MMAE cytotoxin. Therefore, we also studied the sensitivity of different mesothelioma cell types to free MMAE, added as a concentration series (Supplementary Fig. S4). This experiment showed that the MMAE sensitivity of H-Meso-1 cells was indeed markedly lower than that of NCI-Meso79 cells. Other mesothelioma cell lines, ONE58 and JL-1, showed a sensitivity similar to NCI-Meso79 cells, although none of the mesothelioma cells were as sensitive to MMAE as the squamous cell lung carcinoma cell line EBC-1, used as a high-sensitivity positive control (24); see “Discussion”.

Next, we evaluated the potential effect of a combination treatment, using both the anti-uPARAP ADC and a chemotherapeutic for the treatment of NCI-Meso79 cells *in vitro*. For this purpose, we combined 9b7-MMAE ADC with cisplatin, a widely used antineoplastic agent for first-line mesothelioma therapy. Using a concentration series of both drugs, we tested the effect on cell viability of a total of 49 combination conditions. It was evident that additive or synergistic effects could be achieved in certain concentration ranges (Fig. 4B). To assess the concentration dependence of a potential synergistic effect, we used the zero-interaction potency (ZIP) approach (see “Materials and Methods”; ref. 30). Notably, several individual combinations, particularly those in which low concentrations of the ADC were combined with higher cisplatin concentrations (2.5 and 10 μg/mL), demonstrated elevated ZIP synergy values (Fig. 4C; Supplementary Fig. S6A). This was not a unique property of NCI-Meso79 cells, as similar synergistic effects were found in some concentration intervals when combination treatment was studied in the same manner with mesothelioma cell lines such as H-Meso-1 and ONE58, although a third cell line, JL-1, showed little or no synergism (synergy graphs in Supplementary Fig. S5 and corresponding dose response data are given in Supplementary Fig. S6).

**Figure 5.**
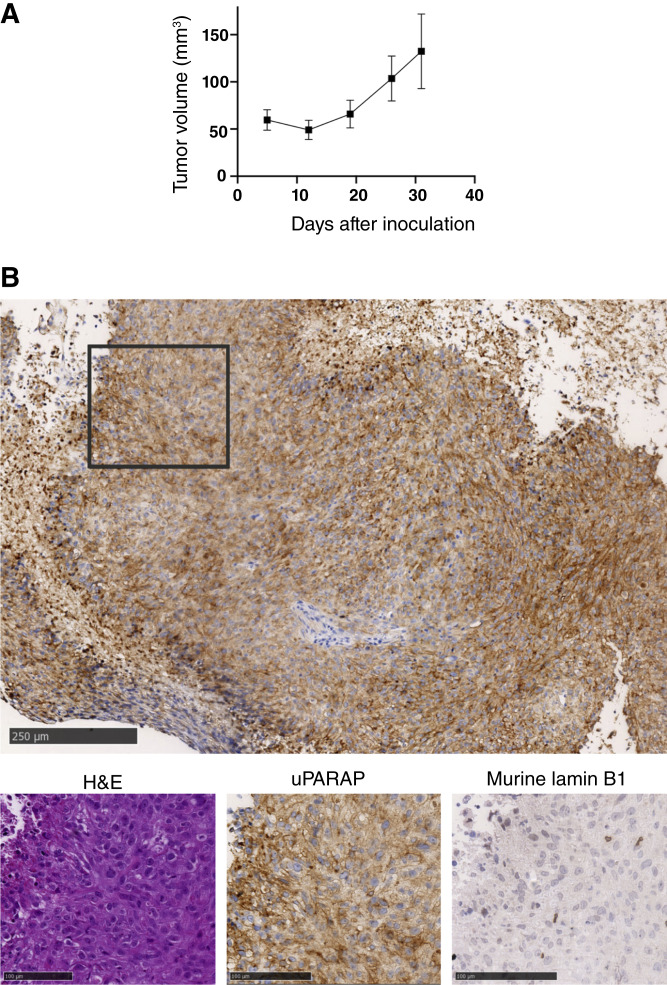
Establishing the NCI-Meso79 tumor model in mice. **A,** Tumor growth curve for NCI-Meso79 subcutaneous tumors in NSG mice (mean ± SD, *n* = 3) before treatment. **B,** Histologic analysis of NCI-Meso79 tumors grown in mice. Example shows a tumor-bearing mouse in the saline-treated group in Fig. 6A. Top, IHC staining of a larger tumor area for uPARAP (scale bar, 250 μm). Staining of the cross-section reveals uniform expression of uPARAP through the tumor tissue. Bottom, enlarged views of a smaller tumor region of adjacent sections (scale bars, 100 μm). Left, H&E staining; middle, IHC staining for uPARAP, demonstrating consistent high-intensity staining; right, mouse-specific lamin B1 staining, showing very low infiltration of mouse fibroblasts.

Upon confirming the sensitivity of NCI-Meso79 cells to these treatment modalities *in vitro*, we established an NCI-Meso79 subcutaneous tumor model in mice for therapy studies *in vivo*. The setup of this model was enabled by a relatively high proliferation rate of these cells in culture and by the finding that they were capable of growing in NSG mice, reaching a treatable tumor volume in about 30 days (tumor growth curve before treatment shown in Fig. 5A).

Histologic analysis of NCI-Meso79 tumors after growth in mice revealed important differences relative to the H-Meso-1 tumors described above. First, the NCI-Meso79 tumors showed a rather uniform distribution of uPARAP expression throughout the tumor tissue [Fig. 5B (top)]. Only minor variations in uPARAP intensity were observed, and no uPARAP-negative cancer cells were identified. Second, the infiltration of host stromal cells, revealed by the species-specific staining of murine lamin B1, was considerably less pronounced than that found in the H-Meso-1 model [Fig. 5B (bottom)].

**Figure 6.**
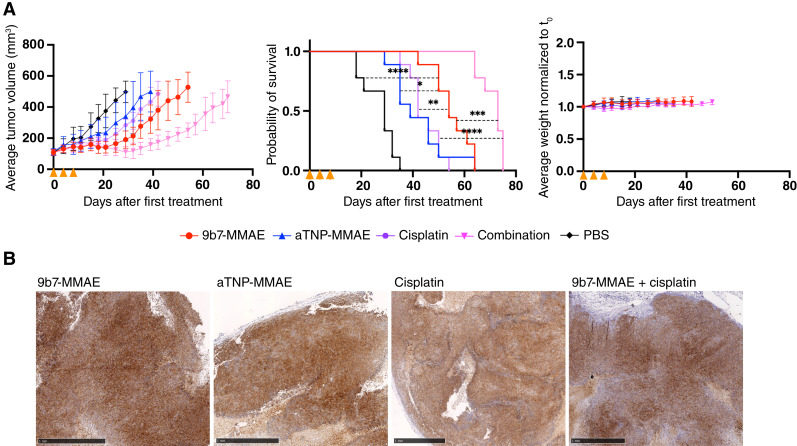
Treatment of NCI-Meso79 tumors in mice using single-reagent and combination therapy. **A,** Tumor development, mouse survival, and mouse weight upon treatment. NCI-Meso79 cells were injected into the flank of NSG mice (*n* = 9 in each treatment group). Tumors were allowed to grow until a volume of 80–150 mm^3^. Randomized treatment groups were treated intravenously with 9b7-MMAE (red) or nontargeted aTNP-MMAE (blue) at 6 mg/kg on days 0, 4, and 7 (orange arrowheads). Cisplatin (purple) was administered intraperitoneally at 1 mg/kg on the same dates in the relevant groups, whereas the combination group (pink) received injections of 6 mg/kg 9b7-MMAE intravenously and 1 mg/kg cisplatin intraperitoneally. PBS-injected (black) mice were used as vehicle control. Left, average tumor volume in groups (curves are shown using the last observation carried forward until 50% of the mice had reached the humane endpoint); middle, Kaplan–Meier curves showing survival for each treatment group with log-rank test (all survival curves being limited by the experiment’s predefined humane endpoints); and right, average mouse weight in groups. Grouped data are shown as the mean ± SD. *, *P* = 0.03; **, *P* = 0.003; ***, *P* = 0.0001; ****, *P* = <0.001. **B,** Representative histologic comparison of uPARAP staining in NCI-Meso79 tumors treated with indicated drugs (for comparison with saline, see Supplementary Fig. S5B). Scale bars, 1 mm. Similar and uniform expression levels were seen in all NCI-Meso79 tumors.

After establishing palpable NCI-Meso79 tumors, a treatment study was performed with ADCs according to the same schedule as used above with H-Meso-1 tumors and with additional treatment arms, including cisplatin and cisplatin plus 9b7-MMAE combination (Fig. 6A). Treatment with the uPARAP ADC had a pronounced antitumor effect, including an initial tumor regression (around d15-d18 after first treatment) and a further delayed growth relative to the three control groups [Fig. 6A (left); individual tumor volume data shown in Supplementary Fig. S7]. A slightly smaller delay was noted after cisplatin treatment. At day 29 (the median survival time for PBS-treated mice), tumor volumes were on average 43% lower in the cisplatin group and 62% lower in the 9b7-MMAE group than in the PBS group. Strikingly, the effect of the combination treatment was markedly stronger than that obtained with either drug alone. The combination treatment reduced the average tumor volume by 78% on day 29, with continued tumor shrinkage thereafter. Both drugs used individually also increased mouse survival, but a stronger effect was again achieved by the combination treatment. Treatment with 9b7-MMAE prolonged the median OS by 25 days (54 vs. 29 days after first treatment, compared with the saline group), whereas the combination treatment led to a median survival of 73 days [Fig. 5A (center)]. As for the H-Meso-1 trial, no effect on the weight of the included mice was observed following treatment with ADCs, cisplatin, or the combination [Fig. 6A (right)].

Histologic analyses after treatment revealed high and homogeneous expression of uPARAP in these tumors, persisting across tumors treated with 9b7-MMAE ADC, aTNP-MMAE ADC, saline, cisplatin, or the cisplatin plus ADC combination (Fig. 6B). Finally, also after treatment, no notable fibroblast infiltration was observed in the NCI-Meso79 tumors.

## Discussion

The work presented here supports the utility of uPARAP as a promising ADC target for treating mesothelioma, a currently incurable disease. Moreover, such ADC treatment may be combined with the platinum-based drugs that are currently used for the standard treatment of this cancer type. The pronounced delay in tumor growth and the prolonged survival of treated mice obtained after combination treatment (Fig. 6) seem remarkable, considering the limited treatment options for this disease.

For the *in vivo* studies in this work, we prepared ADCs comprising an anti-uPARAP antibody, 9b7, and a nontargeted mAb, aTNP, both conjugated to the tubulin inhibitor MMAE, which is a widely used payload in clinically approved ADCs (33). The average DAR obtained, between 4 and 4.5, is consistent with the previously described DAR optimal range in clinical MMAE ADCs (34, 35). When using this anti-uPARAP ADC as a monotherapy *in vivo*, a strong antitumor effect was obtained when using doses of 6 mg/kg.

The need for these relatively high doses was not unexpected in the light of the rather modest sensitivity of the mesothelioma cells to the same ADC *in vitro* (Fig. 4; Supplementary Fig. S3). However, a major factor in this connection seemed to be a relatively low sensitivity of the mesothelioma cells to the free MMAE cytotoxin. This was shown by comparing the MMAE sensitivity of the mesothelioma cells with that of the squamous cell lung carcinoma cell line, EBC-1 (Supplementary Fig. S4), a cell type that does not express uPARAP but has been shown previously to have a high sensitivity to the free cytotoxin (24).

These observations indicate that further improvements may be achieved by studying alternative cytotoxins as the payload component of an anti-uPARAP ADC. To this end, we have previously characterized an anti-uPARAP ADC with the payload PNU159682 (8, 36), demonstrating a high potency against mesothelioma cells *in vitro*. However, the very toxic properties of this payload made it less attractive for *in vivo* studies in this work. On the other hand, a very interesting alternative could be the use of deruxtecan-type topoisomerase I inhibitors (7). This option is particularly relevant as a phase 1/2 clinical trial with a humanized ADC directed against uPARAP, including a payload belonging to this class, has been recently initiated with soft tissue sarcomas as the primary indication (ClinicalTrials.gov ID: NCT06797999). Altogether, additional studies will be required to address the sensitivity of mesothelioma cells to anti-uPARAP ADCs with different payloads, also including studies on factors such as the cellular expression of multidrug resistance genes or other cellular properties that might influence the sensitivity patterns.

Our preclinical work comprised two xenograft mouse models with different properties. One was based on a fast-growing human mesothelioma cell line, H-Meso-1, derived from a 42-year-old patient with pleural biphasic mesothelioma (37). Due to its tumorigenic properties, H-Meso-1 was chosen instead of ONE58 and JL-1 mesothelioma cell lines despite its narrower sensitivity window found previously in experiments with a PNU-based ADC *in vitro* (8).

Whereas the H-Meso-1 xenograft model was initially chosen as a preliminary system to enable an early evaluation before moving on to patient-based isolates, it turned out to demonstrate important properties of the targeting principle in its own right. In this model, uPARAP expression was not restricted to tumor cells. Infiltrating fibroblast-like host cells were abundant in the tumor tissue and consistently expressed the target receptor (Fig. 1B, ROI 2). The targeting of these cells, enabled using a species cross-reactive antibody, likely contributed to obtaining a period of growth arrest and significantly increased survival in the ADC-treated mice using this model (Fig. 2A). Thus, in addition to a direct ADC-mediated eradication, the indirect targeting of fibroblasts likely imposed a bystander effect following treatment with our species cross-reactive ADC containing membrane-permeable MMAE. A similar mechanism has also been shown with certain other ADCs with stromal target expression (38) and with an anti-uPARAP ADC in certain carcinoma models (24). Other factors, including effects of the TME, may also contribute. Such factors could influence intrinsic cell behavior in various ways, including ADC sensitivity.

Irrespective of the mechanism, the stromal contribution was most likely an important factor in the treatment efficiency found with H-Meso-1 tumors *in vivo*. The observed effect on these tumors was also noteworthy, given the substantially heterogeneous uPARAP expression, in which some tumor cell islands were uPARAP-positive whereas others lacked expression (Fig. 1B). A similar heterogeneity will most likely exist in patients with mesothelioma (8).

In the H-Meso-1 treatment experiment, the unconjugated antibody treatment arm was included as a control to reveal any impact of the 9b7 mAb alone. This treatment did not exhibit any antitumor activity, ruling out a receptor-blocking function as the cause of the observed targeted ADC effects. This observation is important, as uPARAP may play different roles in different cancers. For example, prior work has shown that antibody-mediated inhibition of uPARAP in an osteosarcoma mouse model leads to a pronounced protection against tumor-mediated bone degradation, although this occurred without affecting tumor growth (15).

A strong effect of ADC treatment, with a pronounced delay in tumor growth and a markedly increased survival of the treated mice, was also observed in the patient-derived NCI-Meso79 model, which showed a more homogeneous expression of uPARAP on the tumor cells and a lower degree of fibroblast infiltration [Fig. 6A (left and middle)]. This effect was at least as strong as that obtained above in the H-Meso-1 model.

Notably, for both tumor models, the uPARAP expression in remaining tumor cells seemed to be unchanged after ADC treatment (Figs. 1B, 2B, 5B, and 6B). In principle, this lack of downregulation would enable new rounds of treatment to achieve an even higher therapeutic effect.

For the NCI-Meso79 model, we included treatment arms using cisplatin, and the cisplatin combination with ADC, as multimodality treatment regimens with combinatory chemotherapy can show improved OS benefit for patients with mesothelioma (39). This combination both delayed tumor growth and resulted in a survival gain markedly stronger than that obtained with either treatment alone (Fig. 6A).

ADC combinations are a leading treatment option for resistant tumors and are currently under investigation in clinical trials (40). The synergistic or additive effect of individually effective drugs provides a solid foundation for overcoming drug resistance, typically achieved by combining therapeutic agents with different mechanisms of action (41). In our case, cisplatin induces apoptosis by binding to purine N7 sites, causing DNA damage and blocking cell division (42), whereas MMAE achieves this by inhibiting microtubule assembly and tubulin-dependent GTP hydrolysis (43). Our *in vitro* results, which involved 49 combinations of the two drugs to assess potential synergism in the combination treatment, suggested that the combination of these drugs produces an additive effect in some dosage intervals, with a synergistic effect in others. This was analyzed by the ZIP approach (30), which incorporates both of the individual drug potencies and takes their interaction into account. Similar studies with other cultured mesothelioma cells suggested that synergistic effects could be obtained in several, although not all, of these cell lines.

The sum of our *in vitro* and *in vivo* results with the combination treatment opens the possibility that the addition of a uPARAP-targeting ADC to a treatment regimen with cisplatin would allow for reduced dosages in patients. This could decrease the side effects of cisplatin, such as nephrotoxicity and cardiotoxicity (44). As these side effects differ from those of MMAE, which are primarily peripheral neuropathy and neutropenia (45), the combination may prevent clinically prohibitive side effects while maintaining antitumor efficacy. To this end, the combination treatment with cisplatin and 9b7-MMAE ADC seemed to be well tolerated in mice, with no substantial difference in the mean weight of the treated animals in the combination group compared with all other groups [Fig. 6A (right)]. Similar considerations also make it evident that the potential use of anti-uPARAP ADCs with different payloads, as discussed above, could enable additional means of synergism.

The nontargeted ADC (aTNP-MMAE), used as a negative control reagent in our study, was largely without effect in the mouse model with H-Meso-1 cells. In the NCI-Meso79 cohort, treatment with this reagent did lead to some delay in tumor growth, although this effect was clearly lower than that obtained after uPARAP-targeted treatment [Fig. 6A (left)]. A variable effect on tumor growth of nontargeted, MMAE-based ADCs has likewise been observed in other preclinical studies (46, 47). The complete background for this phenomenon remains unclear and likely involves several mechanisms, such as unspecific (extracellular) linker cleavage, macropinocytosis, cellular uptake through Fc receptors or other receptors unrelated to the target under study, or as a consequence of the enhanced permeability and retention effect, which is common in most solid tumors and responsible for target-independent effects of both ADCs and other macromolecular drugs (48–50). The role of many of these effects would likely differ between tumor models. Furthermore, as discussed above, different tumor models display variability in sensitivity to the free cytotoxin that may emerge after unspecific ADC cleavage.

As previously shown, mesothelioma exhibits strong upregulation of the target receptor uPARAP across all primary subtypes, independent of asbestos exposure, neoadjuvant chemotherapy, tumor–node–metastasis staging, or nuclear grade (8). Alongside our current treatment study in xenograft models, this underscores uPARAP as a promising target for ADC-based therapy in this disease. An additional contributing factor is the upregulation of uPARAP on CAFs, as observed in the H-Meso-1 tumor model in the current study. Nevertheless, a decision to implement a uPARAP-directed treatment for patients should likely be based on an individual assessment of uPARAP expression levels.

This study is the first to demonstrate the antitumor activity of uPARAP-targeting ADCs in mesothelioma models *in vivo*. Previously, uPARAP has been studied as a target for ADC therapeutics in mouse models with leukemic and osteosarcoma cells (16, 29), resulting in promising outcomes with minimal side effects. As a phase-1 clinical trial with a humanized anti-uPARAP ADC has now been initiated in a different cancer indication, as mentioned above, this underscores the clinical relevance of our work, which may pave the way for expanding treatment strategies for mesothelioma, a disease with an extraordinary need for novel treatment options.

## Supplementary Material

Supplementary Figure S1 Figure S1. Molecular characterization of ADCs.

Supplementary Figure S2 Figure S2. Individual tumor volume data for H-Meso-1 tumors in the in vivo treatment experiment shown in Fig. 2.

Supplementary Figure S3 Figure S3. Sensitivity of a selection of patient-derived cell isolates to 9b7-MMAE.

Supplementary Figure S4 Figure S4. Sensitivity of mesothelioma cells (H-Meso-1, NCI-Meso79, JL-1, and ONE58), and high-sensitivity positive control cells EBC-1 to free MMAE.

Supplementary Figure S5 Figure S5. 3D graphs representing synergy scores for 9b7-MMAE and cisplatin combinations.

Supplementary Figure S6 Figure S6. Dose response bars for the treatment experiment using combinations of 9b7-MMAE and cisplatin in vitro

Supplementary Figure S7 Figure S7. Individual tumor volume data for NCI-Meso79 tumors in the in vivo treatment experiment shown in Fig. 5.

## Data Availability

Data generated in this study are available upon request from the corresponding author.
