# Supplementary material for: Targeting uPARAP with an Antibody–Drug Conjugate Exhibits Efficacy against Mesothelioma and Synergizes with Cisplatin
Source: Cancer Res Commun. 2026 Jan 16;6(1):130–42. doi: 10.1158/2767-9764.CRC-25-0381 (PMC12810491; doi:10.1158/2767-9764.CRC-25-0381)
Supplement: Supplementary Figure S1 — Figure S1. Molecular characterization of ADCs. [file crc-25-0381_supplementary_figure_s1_suppsf1.pdf]

Supplementary Material

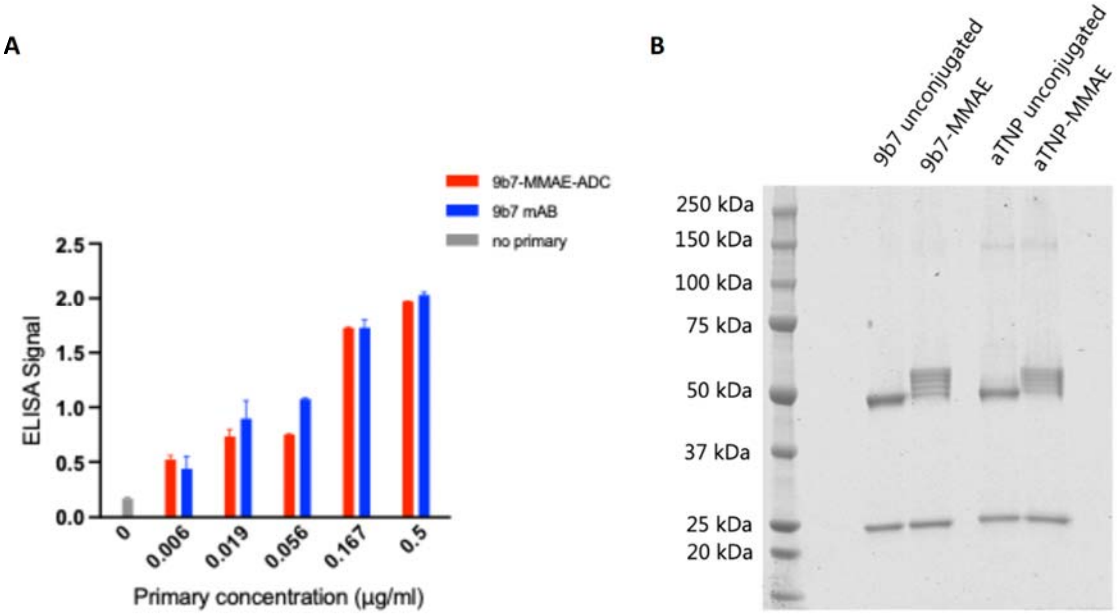

Figure S1. Molecular characterization of ADCs. **A.** Binding of ADC 9b7-MMAE and uncloupled mAb 9b7 to immobilized recombinant uPARAP, measured by ELISA. A comparison of concentration series of the ADC and the uncoupled mAb revealed that they bind with equal affinity to uPARAP. **B.** Electrophoretic analysis of 9b7- and aTNP-MMAE ADCs. Unconjugated antibodies and the ADCs were analyzed by SDS-PAGE under reducing conditions (see Methods; a similar characterization experiment is shown in Ref. 24).
