# Supplementary material for: Targeting uPARAP with an Antibody–Drug Conjugate Exhibits Efficacy against Mesothelioma and Synergizes with Cisplatin
Source: Cancer Res Commun. 2026 Jan 16;6(1):130–42. doi: 10.1158/2767-9764.CRC-25-0381 (PMC12810491; doi:10.1158/2767-9764.CRC-25-0381)
Supplement: Supplementary Figure S2 — Figure S2. Individual tumor volume data for H-Meso-1 tumors in the in vivo treatment experiment shown in Fig. 2. [file crc-25-0381_supplementary_figure_s2_suppsf2.pdf]

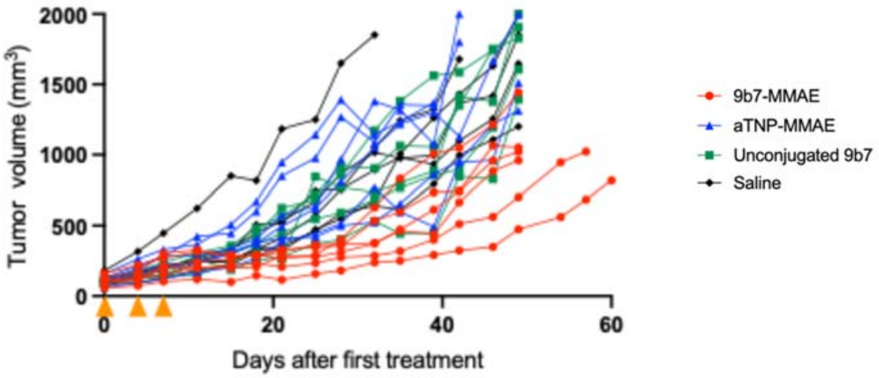

Figure S2. Individual tumor volume data for H-Meso-1 tumors in the *in vivo* treatment experiment shown in Fig. 2. Treatment as follows: 9b7-MMAE (red), non-targeted aTNP-MMAE (blue), or unconjugated mAb 9b7 (green) were administered at 6 mg/kg in i.v. injections on days 0, 4, and 7 (orange triangles). Saline (black) was used as a vehicle control.
