# Supplementary material for: Targeting uPARAP with an Antibody–Drug Conjugate Exhibits Efficacy against Mesothelioma and Synergizes with Cisplatin
Source: Cancer Res Commun. 2026 Jan 16;6(1):130–42. doi: 10.1158/2767-9764.CRC-25-0381 (PMC12810491; doi:10.1158/2767-9764.CRC-25-0381)
Supplement: Supplementary Figure S3 — Figure S3. Sensitivity of a selection of patient-derived cell isolates to 9b7-MMAE. [file crc-25-0381_supplementary_figure_s3_suppsf3.pdf]

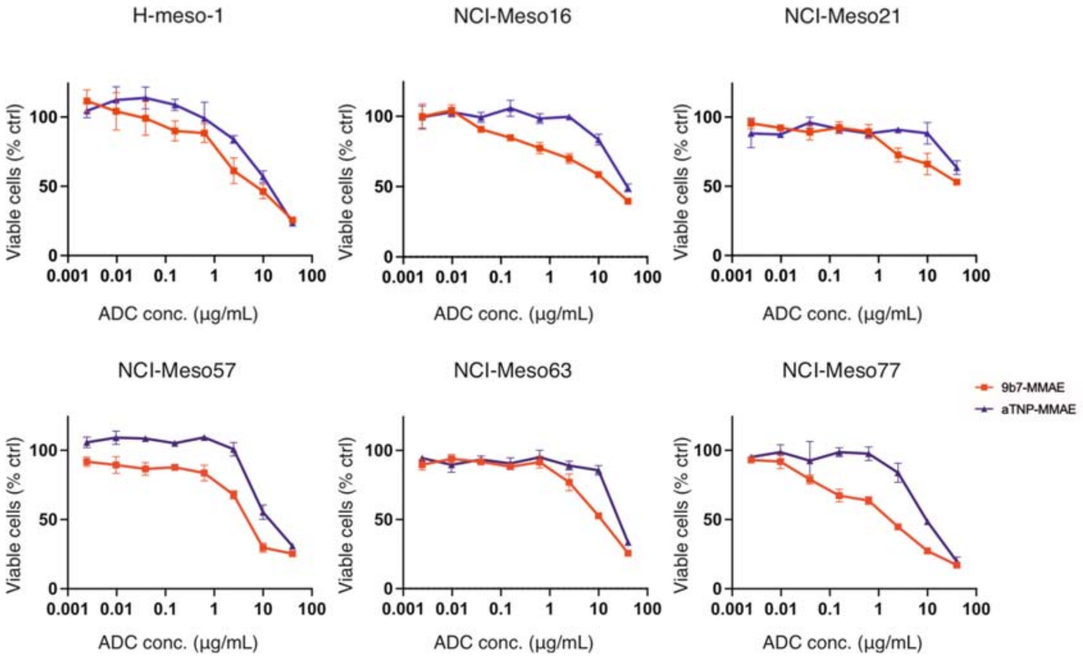

Figure S3. Sensitivity of a selection of patient-derived cell isolates to 9b7-MMAE. The viability of cells after 6 days of cultivation in the presence of varying concentrations of 9b7-MMAE (red) or aTNP-MMAE (blue) was determined in triplicate samples using the MTS assay. H-Meso-1 cells were included in the analysis for comparison. Percentages are presented relative to untreated control cell populations as mean ± SD.
