# Supplementary material for: Targeting uPARAP with an Antibody–Drug Conjugate Exhibits Efficacy against Mesothelioma and Synergizes with Cisplatin
Source: Cancer Res Commun. 2026 Jan 16;6(1):130–42. doi: 10.1158/2767-9764.CRC-25-0381 (PMC12810491; doi:10.1158/2767-9764.CRC-25-0381)
Supplement: Supplementary Figure S4 — Figure S4. Sensitivity of mesothelioma cells (H-Meso-1, NCI-Meso79, JL-1, and ONE58), and high-sensitivity positive control cells EBC-1 to free MMAE. [file crc-25-0381_supplementary_figure_s4_suppsf4.pdf]

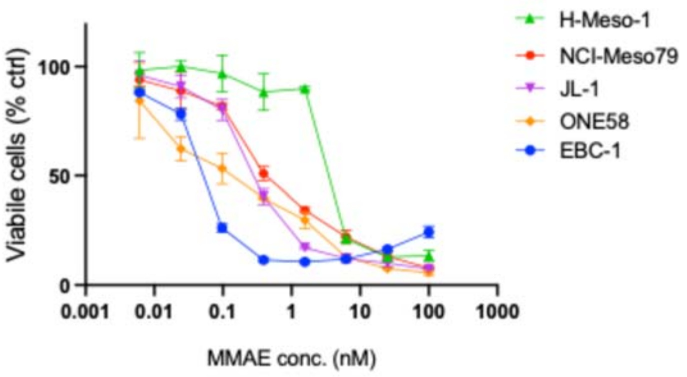

Figure S4. Sensitivity of mesothelioma cells (H-Meso-1, NCI-Meso79, JL-1, and ONE58), and high-sensitivity positive control cells EBC-1 to free MMAE. The MTS assay was used to calculate the viability of cells after 6 days of cultivation. Results are shown relative to DMSO-treated control cell populations and are presented as mean ± SD.
