# Supplementary material for: Targeting uPARAP with an Antibody–Drug Conjugate Exhibits Efficacy against Mesothelioma and Synergizes with Cisplatin
Source: Cancer Res Commun. 2026 Jan 16;6(1):130–42. doi: 10.1158/2767-9764.CRC-25-0381 (PMC12810491; doi:10.1158/2767-9764.CRC-25-0381)
Supplement: Supplementary Figure S5 — Figure S5. 3D graphs representing synergy scores for 9b7-MMAE and cisplatin combinations. [file crc-25-0381_supplementary_figure_s5_suppsf5.pdf]

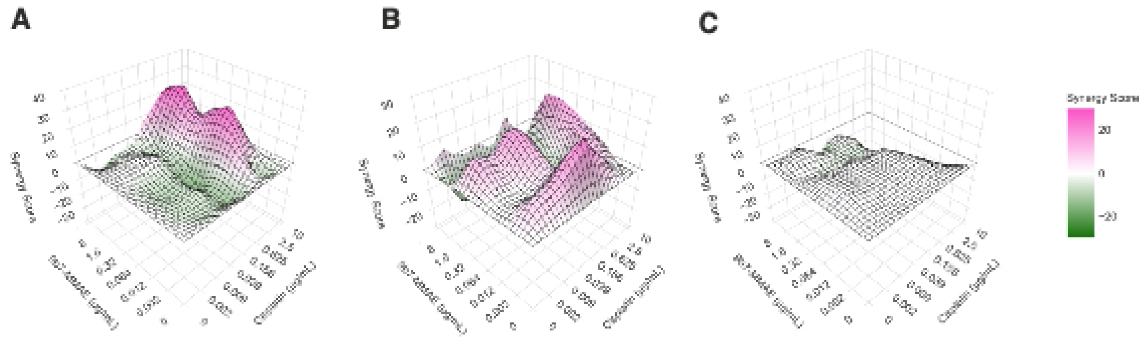

Figure S5. 3D graphs representing synergy scores for 9b7-MMAE and cisplatin combinations for the effect on **A.** H-Meso-1, **B.** ONE58, and **C.** JL-1 cells, according to the zero-interaction potency (ZIP) model. See Fig. 4C for details. 9b7-MMAE concentrations from 0.002 to 40 μg/mL and cisplatin concentrations from 0.002 to 10 μg/mL were analysed in all cases. Dose-response data, along with calculated synergy scores, can be found in Fig. S6.
