# Supplementary material for: Targeting uPARAP with an Antibody–Drug Conjugate Exhibits Efficacy against Mesothelioma and Synergizes with Cisplatin
Source: Cancer Res Commun. 2026 Jan 16;6(1):130–42. doi: 10.1158/2767-9764.CRC-25-0381 (PMC12810491; doi:10.1158/2767-9764.CRC-25-0381)
Supplement: Supplementary Figure S6 — Figure S6. Dose response bars for the treatment experiment using combinations of 9b7-MMAE and cisplatin in vitro [file crc-25-0381_supplementary_figure_s6_suppsf6.pdf]

A

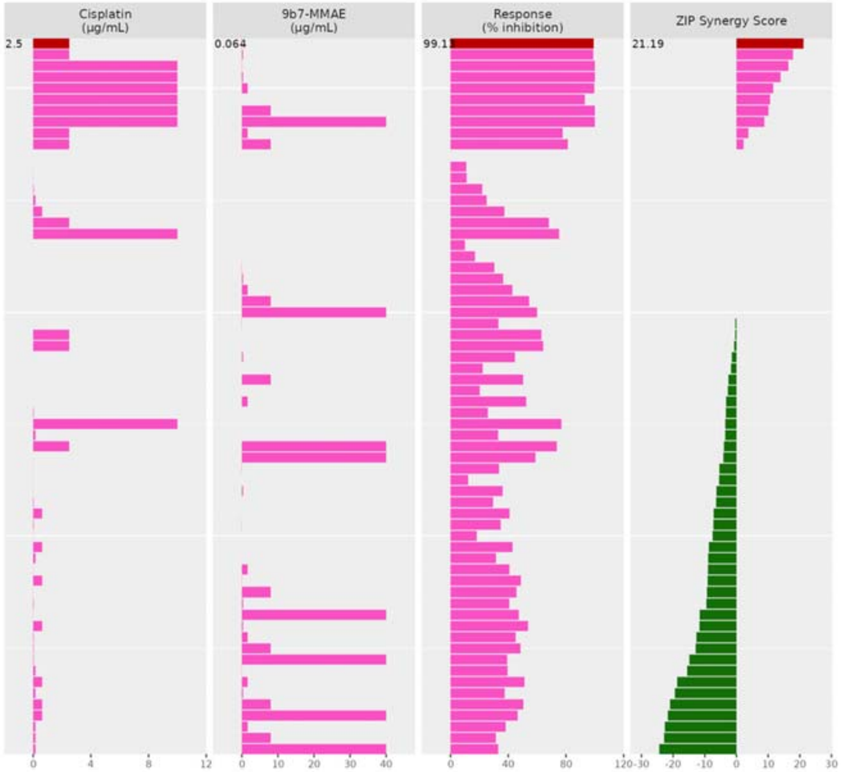

Cisplatin
(μg/mL)
9b7-MMAE
(μg/mL)
Response
(% inhibition)
ZIP Synergy Score
2.5
0.064
99.11
21.19
0
4
8
12
0
10
20
30
40
0
40
80
120
-30
-20
-10
0
10
20
30

B

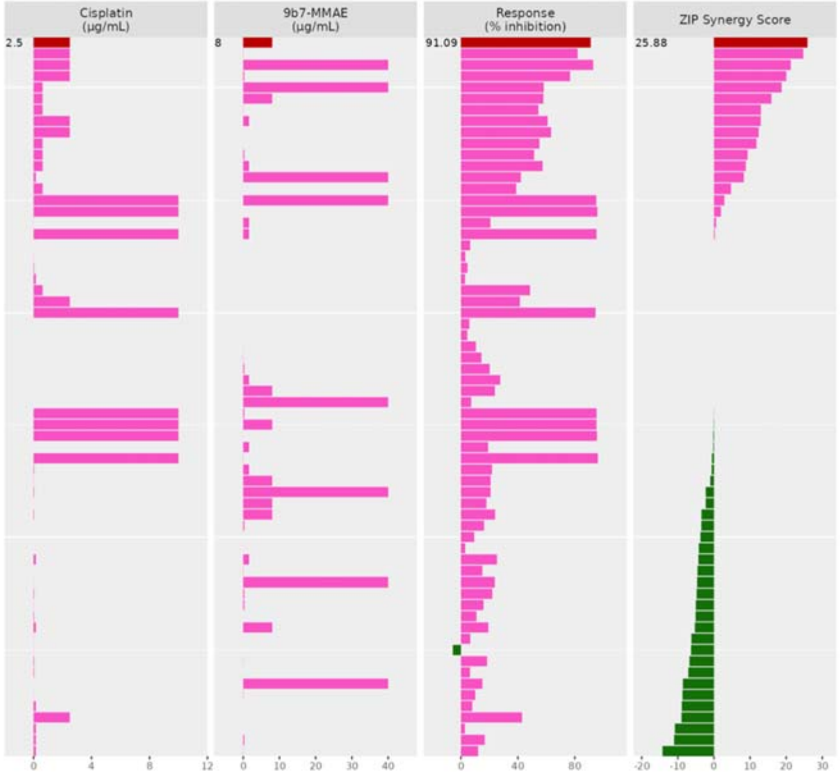

Cisplatin
(μg/mL)
9b7-MMAE
(μg/mL)
Response
(% inhibition)
ZIP Synergy Score
2.5
8
91.09
25.88
0
4
8
12
0
10
20
30
40
0
40
80
-20
-10
0
10
20
30

C

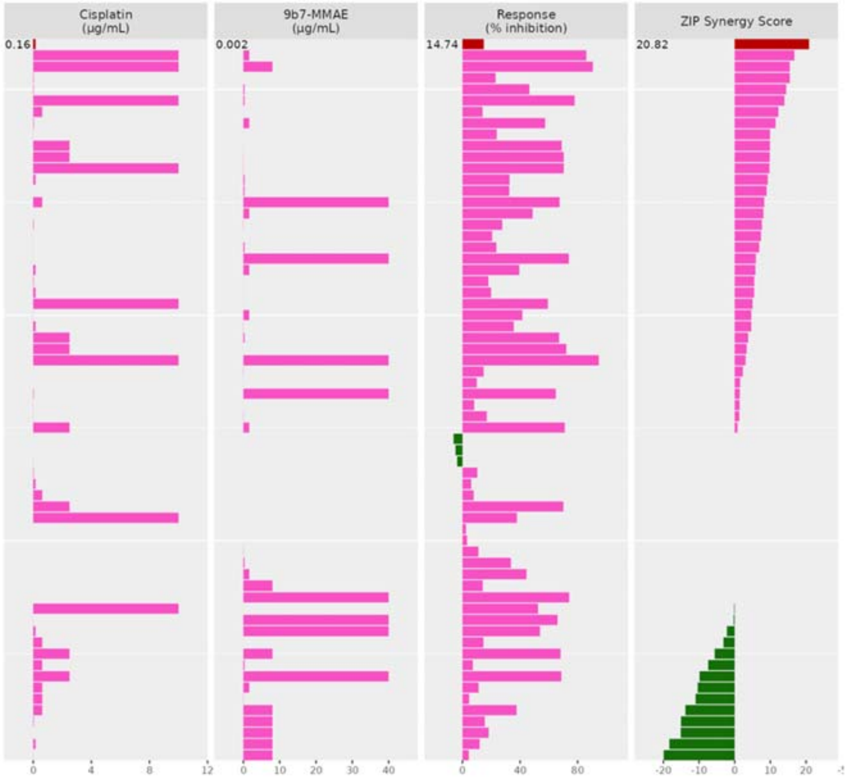
Cisplatin (µg/mL)
9b7-MMAE (µg/mL)
Response (% inhibition)
ZIP Synergy Score
0.16
0.002
14.74
20.82

D

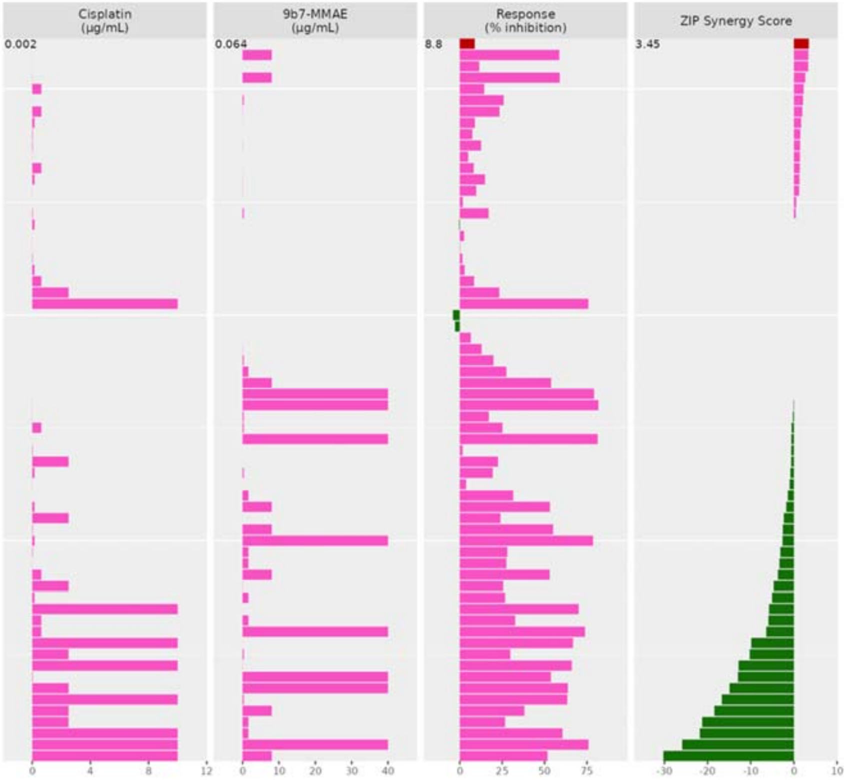
Cisplatin (µg/mL)
9b7-MMAE (µg/mL)
Response (% inhibition)
ZIP Synergy Score
0.002
0.064
8.8
3.45

Figure S6. Dose response bars for the treatment experiment using combinations of 9b7-MMAE and cisplatin *in vitro* for **A.** NCI-Meso79, **B.** H-Meso-1, **C.** ONE58, and **D.** JL-1 cells. The data from the combination and mono treatment series are shown as % inhibition response. The corresponding synergy score (for the combination series) was calculated using the ZIP scoring approach (see Methods section).
