# Supplementary material for: Targeting uPARAP with an Antibody–Drug Conjugate Exhibits Efficacy against Mesothelioma and Synergizes with Cisplatin
Source: Cancer Res Commun. 2026 Jan 16;6(1):130–42. doi: 10.1158/2767-9764.CRC-25-0381 (PMC12810491; doi:10.1158/2767-9764.CRC-25-0381)
Supplement: Supplementary Figure S7 — Figure S7. Individual tumor volume data for NCI-Meso79 tumors in the in vivo treatment experiment shown in Fig. 5. [file crc-25-0381_supplementary_figure_s7_suppsf7.pdf]

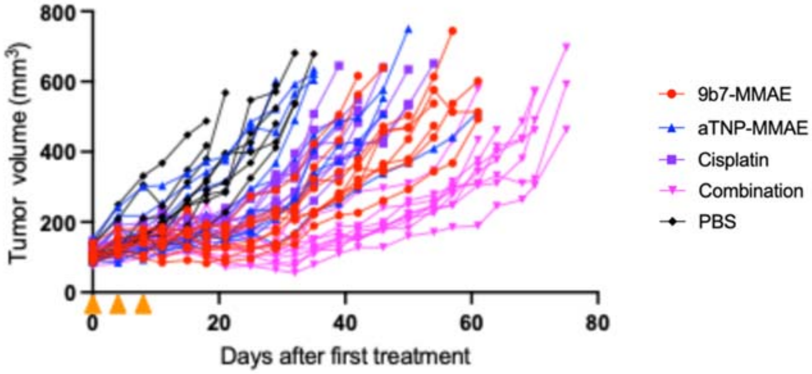

Figure S7. Individual tumor volume data for NCI-Meso79 tumors in the *in vivo* treatment experiment shown in Fig. 5. Treatment as follows: 9b7-MMAE (red) or non-targeted aTNP-MMAE (blue) were administered at 6 mg/kg in i.v. injections on days 0, 4, and 7 (orange triangles). Cisplatin (purple) was administered i.p. at 1 mg/kg, and the combination group (pink) received subsequent injections of 6 mg/kg 9b7-MMAE i.v. and 1 mg/kg cisplatin i.p. PBS (black) was used as a vehicle control.
